# The Merits of Soft and Cohesive Gold as a Filling Material

**Published:** 1882-01

**Authors:** A. A. Blount

**Affiliations:** Geneva, Switzerland.


### ARTICLE IV.

### *The Merits of Soft and Cohesive Gold as Filling Material.*

BY A. A. BLOUNT, M. D., D. D. S., GENEVA, SWITZERLAND.

(Read before the American Dental Society of Europe.)

At a meeting of the American Dental Association held in Boston, the merits of soft and cohesive gold were discussed by many of the prominent dentists in attendance. By a careful perusal of the remarks of these gentlemen, it is plain to be seen that but few of them entertain similar views. One gentleman "thought that a young man who came here to learn how to fill a tooth would go home without knowing how any better than when he came." It is not necessary to recapitulate what was said during this discussion, as every reader of the transactions is already familiar with the views expressed by these gentlemen. In my opinion our greatest failures may be attributed, not to the preparations of gold we have used, but to the style of instruments. I do say, most emphatically, that no dentist who uses cohesive or heavy foils can make as perfect a filling with serrated points, as he who has the same preparation of gold with smooth oval points. One gentleman spoke of the cohesive gold "drawing away from the walls of the cavity," that it would "ball or bur up towards the center." That is not the fault of the gold, but the natural result of the use of serrated instruments and the mallet. Every gold beater will tell you that it is impossible to beat out gold into foil with a serrated hammer. Your hammer must be oval on its face. The same law applies to our instruments. In order to obtain a lateral expansion of the foil, the face of the instruments must be smooth and oval.

I propose to present to this association a method entirely new and original in the manipulation of soft and cohesive foils; a method which I am sure every dentist will admit after a careful and thorough trial, to be the most rational method of filling teeth with these preparations of gold.

We all recognize the fact that *soft* foil packed against the walls of a cavity *will* make a perfect filling, as the contact of the gold with the tooth is *perfect* and *complete.* We all know, as well that *cohesive* foil packed against the walls of a cavity does *not always* make a perfect filling, because we are never *sure* that the contact of the gold with the walls of the cavity is *perfect* and *complete.* We are also aware that a tooth filled entirely with soft foil has not as lasting a filling; that is, it does not wear as well, as one made of cohesive foil. We often see a filling of cohesive foil standing perfect and beautiful as the day it was made, with the walls of the cavity blackened and decayed around it. We see, as well, fillings of soft foil rough and unsightly, and so soft sometimes that we can easily pierce them with an excavator, but yet the tooth presents no signs of further decay.

An experience of nearly forty years in the use of all the recognized methods of filling teeth with the various preparations of gold foils, with as many failures, and, I trust, with an equal share of success, as others, I am free to acknowledge that I never understood the proper method of manipulating soft and cohesive till within the last year.

Taking all the recognized facts as I have stated them above, the idea presented itself to me, why not adopt that which experience has taught, viz.: put *soft foil* against the *walls* of the cavity and fill in with hard or *cohesive foil?* It is very simple and very easily done. There we have the two desirable results which we strive so hard to obtain—*perfect adaptation* to the walls, and a surface that will resist the action of mastication. What more can we wish, and what more can we accomplish?

I will as briefly as possible attempt to describe my method of manipulating. It is impossible, in a brief article, to enter into all the minutiæ or present the various difficulties that we will have to encounter; but our experience and judgment will teach us how to overcome them. It is necessary only that we should have the principle, and then let us apply it according to our light. One important idea, however, should govern us in beginning an operation, viz.: to reduce every difficult filling, as far as possible, to a simple one. This we can best accomplish by filling all the difficult or inaccessible portions of the cavity first, thus leaving only a plain, simple cavity to finish, which we can do easily and rapidly.

We will suppose our cavity to be an approximal one in a molar or bicuspid; we will commence the operation by lining the cavity, beginning at the cervical wall where we have already prepared it in such a manner as to retain the foundation or starting piece of our filling. Let us begin with *soft foil*, and fill this point thoroughly, allowing the gold to extend over or beyond the border; continue this lining of *soft foil* on the lateral walls until we have reached the grinding surface, all the while allowing the gold to extend beyond the borders as in the beginning.

If the cavity at the grinding surface is V shaped, line it also. Then we will place a large pellet against the back of the cavity, packing thoroughly but lightly, which will serve to hold the whole in place, and complete the lining process of *soft foil.* We will use the Varney foot instrument with light blows of the mallet, or any other style of instrument with hand pressure, as we may best accomplish the work, but let the *pressure* be *all* the *time against* the *walls* of the *cavity.*

Now let us begin again at the cervical wall as before, only this time with *cohesive foil* and *smooth, oval pointed* instruments, placing *over* the soft a layer of cohesive foil of sufficient thickness to insure perfect solidity. After having thoroughly covered the soft with the cohesive foil, finish the borders, especially the cervical border completely, as this can be accomplished more easily at this stage of the operation than after the filling is completed. This we can most readily do with the *flat* side of our instrument, driving the gold over the edge of the enamel, burnishing it down in such a manner as to insure a complete adaptation of the gold to the edges of the cavity. Now we have completed the most difficult part of the operation, leaving only a plain, simple cavity to fill which may be done very rapidly, as we have no frail walls to retard our progress, they being already covered and protected. We may use pellets, cylinders, blocks, or any preparation of gold that will fill most rapidly and give the hardest surface and the greatest resistance to mastication.

During the whole of the operation let the pressure be, as in the beginning, *against* the *walls* of the cavity, however frail they may be. The soft foil yields under the pressure of the hard, and thus prevents the danger of fracture and at the same time gives support and strength to the frail enamel. I present for your inspection a few specimens of teeth, with the lining of soft and cohesive foil, together with the smooth pointed instruments which will give you a better idea of the method than my imperfect description. One of the specimens, you will observe, is filled on the buccal surface, the lining being of *tin foil*

filled in with hard gold. This combination of metals without the use of mercury, by some chemical process, possesses the peculiar power of hardening, or calcifying the soft and chalky enamel and dentine we so often find in cavities in teeth of young persons. This manner of combining tin and gold will give the same results as that recommended by him whom we all take pleasure in honoring, the pioneer of American dentists in Europe, our much esteemed friend, Dr. Abbot, of Berlin.

